# Impact of chlorine dioxide (ClO_2_) on reverse osmosis (RO) membrane integrity: degradation behavior, surface property changes, and implications for biofouling control

**DOI:** 10.1039/d5ra09010e

**Published:** 2026-07-21

**Authors:** Isni Arliyani, Hilmi Iqlima Khoirunnisa, Heri Septya Kusuma, Muhammad Roil Bilad, Abdulfatah Abdu Yusuf, Muhammad Imam Ammarullah

**Affiliations:** a Department of Environmental Engineering, Faculty of Civil, Planning and Geo Engineering, Institut Teknologi Sepuluh Nopember Surabaya 60111 East Java Indonesia isniarliyani@its.ac.id; b Department of Chemical Engineering, Faculty of Industrial Technology, Universitas Pembangunan Nasional “Veteran” Yogyakarta Yogyakarta 55283 Central Java Indonesia; c Department of Chemical and Process Engineering, Faculty of Integrated Technologies, Universiti Brunei Darussalam Tungku Link BE 1410, Gadong Brunei Darussalam; d Bioengineering and Environmental Sustainability Research Centre, University of Liberia Monrovia Montserrado 1000 Liberia imamammarullah@gmail.com; e Department of Mechanical Engineering, College of Engineering, University of Liberia Monrovia Montserrado 1000 Liberia; f Department of Mechanical Engineering, Faculty of Engineering, Universitas Diponegoro Semarang 50275 Central Java Indonesia

## Abstract

Chlorine dioxide (ClO_2_) has attracted considerable attention as an alternative disinfectant for reverse osmosis (RO) pretreatment because of its broad-spectrum antimicrobial activity, effectiveness over a wide pH range, and lower formation of regulated disinfection by-products compared with conventional chlorination. These characteristics make ClO_2_ a promising strategy for mitigating microbial growth and supporting biofouling control in RO systems. However, the strong oxidative nature of ClO_2_ also presents a significant challenge, as prolonged exposure may induce degradation of the polyamide selective layer, resulting in deterioration of membrane integrity and filtration performance. This study investigated the effects of ClO_2_ concentration and solution pH on the degradation behavior and surface property changes of thin-film composite (TFC) polyamide RO membranes, with particular emphasis on their implications for biofouling control. Membrane performance and structural changes were evaluated using permeate flux measurements, scanning electron microscopy coupled with energy-dispersive X-ray spectroscopy (SEM-EDX), and contact angle analysis. The results demonstrated that increasing ClO_2_ concentration and alkaline conditions accelerated membrane degradation, as evidenced by increased permeate flux, pronounced surface deformation and cracking, disruption of the selective polyamide layer, changes in surface elemental composition, and significant alterations in wettability. The membrane exposed to 0.5 ppm ClO_2_ at pH 6 exhibited the least structural damage and maintained surface characteristics closest to those of the untreated membrane, indicating that this condition provided the most favorable balance between membrane preservation and operational performance. In contrast, exposure to higher ClO_2_ concentration and alkaline pH resulted in severe oxidative deterioration, which compromised membrane integrity despite the potential operational benefits associated with oxidant application. Although biofouling was not directly evaluated through microbial adhesion or biofilm formation experiments, the observed changes in membrane morphology, permeability, and wettability provide valuable insight into surface characteristics associated with fouling propensity. Overall, the findings highlight the importance of optimizing ClO_2_ dosage and operating pH to minimize oxidative membrane degradation while maintaining its practical advantages as a disinfectant in RO pretreatment, thereby providing guidance for improving membrane durability and supporting sustainable membrane-based water treatment.

## Abstract 2

Overall, the findings highlight the importance of optimizing ClO_2_ dosage and operating pH to minimize oxidative membrane degradation while maintaining its practical advantages as a disinfectant in RO pretreatment.

## Graphical abstract

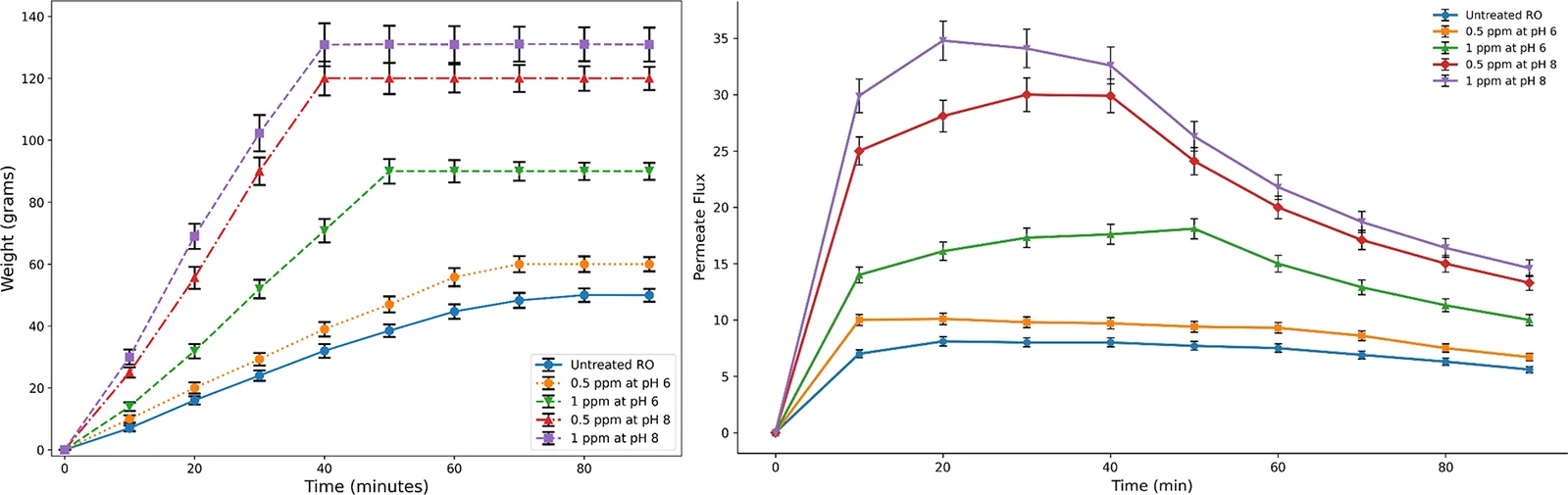

## 1. Introduction

Reverse osmosis (RO) has become one of the most widely adopted membrane-based technologies for producing potable and industrial-grade water^1^ owing to its exceptional ability to remove dissolved salts,^2^ organic compounds,^3^ microorganisms,^4^ and other contaminants from diverse water sources. In Indonesia, the increasing dependence on groundwater as a primary water resource, driven by rapid population growth and industrial development, has intensified the demand for efficient and sustainable water treatment technologies. However, excessive groundwater exploitation has resulted in both quantitative depletion and deterioration of water quality, further emphasizing the importance of advanced purification systems. Despite its high separation efficiency, the long-term operation of RO systems is frequently constrained by membrane fouling, including organic fouling,^5^ inorganic scaling,^6^ particulate deposition,^7^ and particularly biofouling,^8^ which is generally regarded as the most persistent and difficult form of fouling to mitigate.^9^ These fouling phenomena increase hydraulic resistance,^10^ reduce permeate productivity and salt rejection,^11^ elevate operating pressure and energy consumption,^12^ accelerate membrane aging,^13^ and ultimately shorten membrane service life.^14^ Consequently, effective pretreatment strategies are indispensable for minimizing foulant accumulation and preserving membrane performance. Conventional RO pretreatment commonly incorporates chemical conditioning using disinfectants,^15^ coagulants,^16^ antiscalants,^17^ and reducing agents.^18^ Among these, sodium bisulfite (NaHSO_3_) is widely employed to quench residual oxidants prior to membrane exposure,^19^ thereby protecting oxidation-sensitive polyamide active layers from chemical degradation.

Among the various disinfectants employed in water treatment, chlorine dioxide (ClO_2_) has emerged as a promising alternative to conventional chlorination^20^ because of its strong oxidative capability,^21^ broad-spectrum antimicrobial activity,^22^ and relatively low formation of regulated disinfection by-products,^23^ such as trihalomethane (THM) and haloacetic acid (HAA). Unlike free chlorine, ClO_2_ maintains effective disinfection over a broad pH range (approximately pH 4–10)^24^ without promoting significant microbial resistance,^25^ making it particularly attractive for RO pretreatment applications. Furthermore, ClO_2_ facilitates the oxidation and precipitation of dissolved metals,^26^ including iron and manganese, thereby reducing the risk of inorganic fouling and scaling within membrane systems. In practical RO operation, especially in first-pass configurations, ClO_2_ has demonstrated considerable potential for suppressing microbial attachment and delaying biofilm development,^27^ thereby contributing to improved operational stability and reduced cleaning frequency. Nevertheless, these operational advantages are accompanied by an important limitation. Because polyamide thin-film composite (TFC) membranes are intrinsically susceptible to oxidative attack,^28^ prolonged or excessive exposure to ClO_2_ can induce irreversible chemical degradation through oxidation and hydrolysis of the polyamide active layer.^29^ Such degradation may alter membrane morphology,^30^ increase water permeability,^31^ reduce salt rejection,^32^ and ultimately compromise membrane integrity and operational lifespan.^33^ Therefore, successful implementation of ClO_2_ in RO pretreatment requires careful optimization of oxidant concentration and operating conditions to achieve an appropriate balance between effective microbial control and long-term membrane durability.

Previous investigations have substantially advanced the understanding of oxidant-induced degradation of polyamide RO membranes and the development of oxidation-resistant membrane materials. For example, Alayemieka and Lee^34^ demonstrated that ClO_2_ exposure under different pH conditions alters the surface chemistry and morphology of polyamide membranes, leading to changes in permeability and separation performance. Likewise, Verbeke *et al.*^35^ comprehensively described the mechanisms governing oxidative degradation of polyamide membranes and highlighted the critical influence of oxidant concentration, exposure duration, and solution chemistry on membrane stability. Alhoshan *et al.*^36^ and Shukla *et al.*^37^ reported that incorporating zinc-based metal–organic framework (Zn-MOF) nanoparticles into membranes significantly enhances hydrophilicity, surface charge characteristics, water permeability, and antifouling performance. Similarly, nanocomposite membrane technologies have demonstrated improved chlorine resistance and greater resistance to surface fouling as shown by Rameesha *et al.*^38^ Despite these important developments, existing studies have predominantly focused either on improving intrinsic membrane materials or presenting the fundamental mechanisms of oxidative degradation. Comparatively little attention has been devoted to systematically correlating application-relevant ClO_2_ exposure conditions with simultaneous changes in membrane degradation behavior, surface morphology, wettability, and filtration performance. Furthermore, although ClO_2_ is widely recognized for its role in mitigating biofouling during RO pretreatment, relatively few studies have examined how membrane degradation induced by ClO_2_ exposure may alter surface characteristics that influence fouling propensity and, consequently, the practical implications for biofouling control.

Despite these advances, several important research gaps remain. Existing studies have largely investigated either the chemical degradation mechanisms of polyamide membranes under oxidative conditions or the development of advanced membrane materials with improved oxidant resistance. Consequently, limited attention has been given to systematically evaluating how practical operating parameters, particularly ClO_2_ concentration and solution pH, simultaneously influence membrane degradation behavior, surface characteristics, and functional performance under conditions relevant to RO operation. Moreover, many previous investigations have relied on a single analytical technique, focusing either on permeability changes, morphological observations, or chemical characterization in isolation. Such approaches provide only a partial understanding of membrane deterioration and often fail to establish clear relationships between oxidative exposure conditions, structural degradation, surface wettability, and membrane performance. Furthermore, although the application of ClO_2_ is widely recognized for its effectiveness in mitigating microbial growth in RO pretreatment systems, relatively few studies have examined how ClO_2_-induced changes in membrane integrity and surface properties may influence conditions associated with fouling development. Establishing these relationships is essential for optimizing ClO_2_ application while minimizing adverse effects on membrane durability and long-term operational performance.

To address these limitations, the present study investigates the influence of ClO_2_ exposure on the integrity of TFC polyamide RO membranes under controlled oxidant concentrations and pH conditions representative of practical water treatment applications. Rather than directly evaluating biofouling through microbiological or biofilm analyses, this work focuses on showing the degradation behavior of the membrane and the associated changes in surface properties that are relevant to fouling propensity. An integrated experimental approach combining permeate flux measurements, scanning electron microscopy coupled with energy-dispersive X-ray spectroscopy (SEM-EDX), and contact angle analysis is employed to establish relationships between oxidative exposure conditions, structural deterioration, surface morphology, wettability, and membrane performance. Through correlating these complementary performance and characterization techniques, this study provides a comprehensive assessment of how ClO_2_-induced oxidative degradation alters membrane integrity and surface characteristics. The findings further provide practical insights into the operational implications of ClO_2_ application for biofouling control, enabling a more balanced evaluation of its benefits as a disinfectant against its potential impact on membrane durability.

Accordingly, this study aims to evaluate the effects of ClO_2_ exposure on the degradation behavior, surface property changes, and functional performance of RO polyamide membranes under different oxidant concentrations and pH conditions. Specifically, the study seeks to (i) determine the influence of ClO_2_ concentration and pH on membrane degradation and permeate flux, (ii) characterize structural and elemental changes in the membrane surface using SEM-EDX, (iii) evaluate alterations in membrane wettability through contact angle measurements, and (iv) identify operating conditions that minimize membrane degradation while maintaining practical applicability for RO pretreatment. To guide the experimental investigation, four hypotheses are proposed: (H1) increasing ClO_2_ concentration (0.5–1.0 ppm) enhances oxidative degradation of the polyamide selective layer, leading to increased permeate flux due to reduced membrane selectivity; (H2) alkaline conditions (pH 8) accelerate membrane degradation relative to near-neutral conditions (pH 6) by increasing the susceptibility of the polyamide layer to oxidation and hydrolysis; (H3) structural deterioration observed by SEM, including cracking, pore exposure, and disruption of the selective layer, is accompanied by measurable changes in surface wettability as reflected by contact angle measurements; and (H4) the combined effects of ClO_2_ concentration and pH produce distinct trends in membrane degradation, surface properties, and performance, thereby enabling identification of operational conditions that balance membrane integrity with the practical application of ClO_2_ in RO pretreatment systems.

## 2. Materials and methods

### 2.1. Reagents and materials

All chemicals used in this study were of analytical or reagent grade and were used as received without further purification. ClO_2_ stock solution (2.0%, stabilized aqueous solution; Sigma-Aldrich, Merck KGaA, Darmstadt, Germany; Cat. no. 372457) was employed as the oxidizing agent to simulate disinfectant exposure during RO pretreatment. Unlike laboratory-generated ClO_2_ produced through chlorine–chlorite or acid–chlorite reactions, the ClO_2_ used in this study was supplied as a commercially stabilized aqueous solution. According to the manufacturer's specifications, the stabilized formulation contains controlled concentrations of chlorite (ClO_2_^−^) and chlorate (ClO_3_^−^) as typical accompanying species resulting from ClO_2_ stabilization. This commercially available formulation was selected to better represent ClO_2_ products commonly used in practical water treatment and membrane pretreatment applications.

Working ClO_2_ solutions with nominal concentrations of 0.5 and 1.0 ppm were prepared immediately before each experiment by diluting the certified stock solution with tap water to obtain an 800 mL test solution. These concentrations were selected to represent conservative ClO_2_ dosages commonly applied in drinking water treatment and RO pretreatment processes, where low oxidant concentrations are preferred to achieve microbial control while minimizing oxidative degradation of TFC polyamide membranes. The prepared solutions served as model oxidizing environments for evaluating membrane degradation behavior and changes in surface properties under application-relevant operating conditions.

Solution pH was adjusted using a 0.1 M phosphate buffer prepared from potassium dihydrogen phosphate (KH_2_PO_4_; Sigma-Aldrich, Cat. no. P5655) and dipotassium hydrogen phosphate (K_2_HPO_4_; Sigma-Aldrich, Cat. no. P3786). Minor pH adjustments were subsequently performed using sodium hydroxide pellets (NaOH; Merck, Germany; Cat. no. 106462) and hydrochloric acid (HCl, 37%; Merck, Germany; Cat. no. 100317) to achieve the target experimental pH values. The phosphate buffer system was selected because it provides stable pH control while minimizing interference with the oxidative reactions occurring between ClO_2_ and the polyamide membrane during immersion.

Commercial TFC RO membranes (Model TM820C-400, Toray Industries, Japan) were used as membrane specimens throughout this investigation. The membrane consists of a polyamide selective layer supported by a porous polysulfone intermediate layer and a polyester nonwoven backing, representing the membrane architecture most widely employed in full-scale RO water treatment systems. Square membrane coupons measuring 5 cm × 5 cm were prepared from the commercial membrane sheets and used for all degradation experiments and subsequent characterization analyses. Prior to scanning electron microscopy (SEM) observation, membrane surfaces were coated with a thin platinum layer using a platinum sputter target (Ted Pella Inc., Redding, CA, USA; Cat. no. 88941; 99.99% purity) to improve electrical conductivity and image quality during electron beam analysis. The specifications, suppliers, catalogue numbers, and analytical grades of all chemicals and materials employed in this study are summarized in Table 1.

**Table 1:** List of reagents and materials used in this study

Reagent/material	Amount or concentration used	Purpose/description	Supplier	Catalogue number	Purity/grade
ClO_2_ stock solution	Prepared to 0.5 ppm and 1.0 ppm in 800 mL tap water	Simulated disinfection treatment for RO membranes	Sigma-Aldrich (Merck KGaA, Darmstadt, Germany)	Cat. no. 372457	2.0% (stabilized aqueous solution)
KH_2_PO_4_	Used to prepare 0.1 M phosphate buffer (pH 6–8)	pH adjustment of test solution	Sigma-Aldrich (Merck)	Cat. no. P5655	≥99.0% (ACS reagent grade)
K_2_HPO_4_	Used to prepare 0.1 M phosphate buffer (pH 6–8)	pH adjustment of test solution	Sigma-Aldrich (Merck)	Cat. no. P3786	≥99.0% (ACS reagent grade)
NaOH	As required for pH calibration	Adjusting solution pH	Merck (Germany)	Cat. no. 106462	≥98% pellets
HCl 37%	As required for pH calibration	Adjusting solution pH	Merck (Germany)	Cat. no. 100317	Analytical reagent grade
RO TFC	Coupons of 5 cm × 5 cm	Membrane specimens for testing	Toray Industries, Japan	Model: TM820C-400	TFC polyamide
Platinum sputter target	Thin coating for SEM imaging	Conductive coating of membrane surface	Ted Pella Inc. (Redding, CA, USA)	Cat. no. 88941	Pt, 99.99% purity

The working ClO_2_ concentrations used in this study were calculated by dilution of the certified stock solution supplied by the manufacturer. No independent analytical verification of ClO_2_ concentration was performed after solution preparation or during the membrane immersion period. Consequently, gradual decomposition of ClO_2_ and the formation of secondary chlorine-containing species, including chlorite and chlorate, may have occurred throughout the exposure period. Because the objective of this study was to evaluate membrane degradation under practical oxidant exposure conditions rather than to investigate ClO_2_ decomposition kinetics, the nominal concentrations prepared from the certified stock solution were used as the experimental basis. This limitation should therefore be considered when interpreting the relationship between oxidant exposure and membrane degradation behavior.

### 2.2. Preparation of ClO_2_ test solutions

ClO_2_ test solutions were prepared immediately before each experiment by diluting the commercially stabilized 2.0% ClO_2_ stock solution with tap water to obtain working concentrations of 0.5 and 1.0 ppm in a total solution volume of 800 mL.^39^ Tap water was selected as the preparation medium to provide a representative aqueous environment for evaluating membrane exposure under conditions relevant to practical RO pretreatment rather than highly purified laboratory conditions. Following preparation of the ClO_2_ solution, the solution pH was adjusted to the desired value using a 0.1 M phosphate buffer system composed of KH_2_PO_4_ and K_2_HPO_4_. Fine adjustments were subsequently performed using NaOH or HCl until the target pH values were achieved. The preparation procedure was conducted immediately prior to membrane immersion to minimize ClO_2_ loss associated with its natural decomposition in aqueous solution.

The selected ClO_2_ concentrations (0.5 and 1.0 ppm) were chosen to represent a practical dosing range commonly employed in drinking water treatment and RO pretreatment applications. In full-scale membrane systems, ClO_2_ is typically applied at relatively low concentrations to suppress microbial activity while minimizing oxidative deterioration of the polyamide selective layer. Concentrations exceeding this range are generally avoided because prolonged exposure to stronger oxidant levels can accelerate membrane degradation and reduce operational lifespan. Consequently, the selected concentrations were intended to represent conservative yet operationally relevant conditions suitable for evaluating the balance between oxidant exposure and membrane integrity, rather than extreme laboratory exposure scenarios designed to induce rapid membrane failure.

The investigated pH values (pH 6 and pH 8) were selected to represent near-neutral and mildly alkaline conditions frequently encountered in groundwater-fed RO systems and membrane pretreatment processes.^40^ These conditions are particularly relevant because feed water pH is commonly adjusted within this range to optimize disinfection efficiency, reduce scaling potential, and maintain stable membrane operation. Furthermore, solution pH is known to influence the oxidative reactivity of ClO_2_ and the chemical stability of polyamide membranes, thereby affecting membrane degradation behavior and associated changes in surface properties. Evaluating both pH conditions therefore enables assessment of membrane responses under realistic operational environments while providing insight into the combined influence of oxidant concentration and solution chemistry on membrane integrity.

The experimental design intentionally focused on a relatively narrow range of ClO_2_ concentration and pH to simulate practical operating conditions encountered in RO pretreatment systems. This approach allows the observed membrane degradation behavior, permeability changes, and surface property modifications to be interpreted within the context of realistic plant operation rather than highly aggressive chemical exposure conditions. Such an experimental framework is consistent with the objective of the present study, which is to evaluate the effects of operationally relevant ClO_2_ exposure on membrane integrity and surface characteristics and to provide practical implications for biofouling control through optimized disinfectant application. The selected exposure conditions therefore enable systematic comparison of oxidative effects while maintaining direct relevance to the operational management of ClO_2_ in membrane-based water treatment systems.

### 2.3. Experimental design for membrane degradation

The experimental design was developed to systematically investigate the combined effects of ClO_2_ concentration and solution pH on the degradation behavior and surface property changes of TFC polyamide RO membranes. The study employed an immersion-based degradation approach to simulate prolonged oxidant exposure that may occur during RO pretreatment and disinfection processes. With independently varying ClO_2_ concentration and pH within an operationally relevant range, the experimental design enabled the influence of each parameter on membrane integrity to be comparatively evaluated while maintaining conditions representative of practical water treatment applications.

Commercial TFC polyamide RO membranes were cut into square coupons measuring 5 cm × 5 cm using a clean stainless-steel cutter to ensure uniform sample dimensions. Membrane coupons were prepared from representative regions of the membrane sheet while avoiding damaged edges and visible defects that could influence degradation behavior or subsequent characterization. Prior to the immersion experiments, the membrane specimens were visually inspected to ensure surface uniformity and consistency among all samples.

Each membrane coupon was completely immersed in 800 mL of the prepared ClO_2_ solution and maintained under static conditions for 24 h at room temperature to provide sufficient contact time for oxidative interaction between ClO_2_ and the polyamide selective layer. The immersion duration was selected based on previously reported membrane degradation studies employing ClO_2_ exposure under controlled laboratory conditions. Following the exposure period, the membrane coupons were thoroughly rinsed with deionized water to remove residual ClO_2_ and any loosely attached oxidation by-products remaining on the membrane surface. The rinsed membranes were subsequently dried in a laboratory oven at 40–60 °C for 48 h before further membrane performance evaluation and surface characterization.^41^ This post-treatment procedure ensured that residual moisture did not interfere with permeate flux measurements, SEM, or contact angle analyses.

Four membrane exposure conditions together with an untreated control membrane were investigated to evaluate the individual and combined influences of ClO_2_ concentration and solution pH. The experimental matrix is summarized in Table 2. The selected experimental conditions were designed to evaluate membrane degradation under conservative oxidant exposure representative of practical RO pretreatment rather than accelerated ageing under highly aggressive chemical environments. The combination of two ClO_2_ concentrations and two pH conditions enabled comparative assessment of the individual and interactive effects of oxidant dosage and solution chemistry on membrane degradation, permeability, and surface characteristics. The untreated control membrane served as the baseline for evaluating changes induced by oxidative exposure.

**Table 2:** Experimental matrix for membrane degradation study

Sample	ClO_2_ concentration (ppm)	Solution pH	Experimental purpose
Control	0.0	Native tap water	Untreated reference membrane
A	0.5	6	Effect of low ClO_2_ concentration under near-neutral conditions
B	1.0	6	Effect of increased ClO_2_ concentration under near-neutral conditions
C	0.5	8	Combined effect of low ClO_2_ concentration and mildly alkaline conditions
D	1.0	8	Combined effect of high ClO_2_ concentration and mildly alkaline conditions

The scope of the present study was limited to the evaluation of membrane degradation behavior and associated surface property changes following ClO_2_ exposure. Control experiments using pH-adjusted solutions without ClO_2_ were not included in the experimental design; consequently, the independent effect of solution pH cannot be completely separated from the combined influence of pH and oxidant exposure. Furthermore, each experimental condition was conducted using a single independently treated membrane specimen (*n* = 1) because of experimental and resource constraints. Accordingly, the results are presented as comparative observations without statistical analyses such as mean values, standard deviations, or significance testing. Nevertheless, the integrated interpretation of permeate flux measurements, SEM-EDX characterization, and contact angle analysis provides a consistent qualitative assessment of membrane degradation behavior and surface property changes under the investigated operating conditions.

### 2.4. Analysis of RO membrane performance

The hydraulic performance of the RO membranes following ClO_2_ exposure was evaluated using a laboratory-scale cross-flow filtration system. Permeate flux measurements were performed to assess changes in membrane permeability resulting from oxidative degradation of the polyamide selective layer. Because deterioration of membrane integrity is generally accompanied by increased water transport through the membrane, permeate flux was employed as one of the principal performance indicators for evaluating the effects of ClO_2_ concentration and solution pH on membrane degradation.

**Fig. 1:**
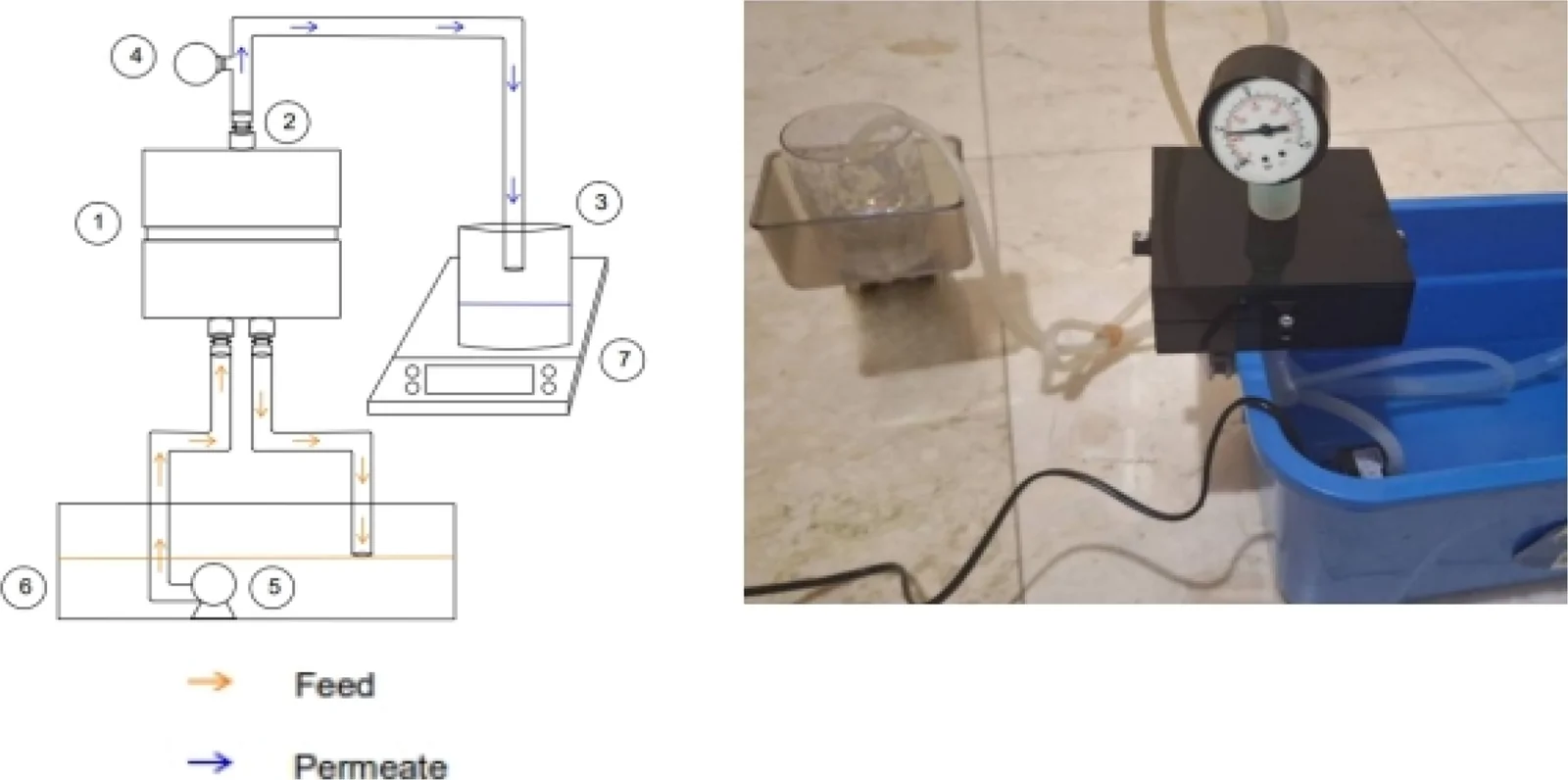
Cross flow filtration system.

Prior to each filtration experiment, the treated membrane coupon was securely installed in the permeation cell of the cross-flow filtration reactor to ensure leak-free operation. The membrane was then compacted under an operating pressure of 65 psi to stabilize membrane performance before data collection. Following compaction, 1 L of feed solution was circulated through the cross-flow system at a flow rate of 1 L min^−1^ for a filtration period of 1 h. Throughout the experiment, permeate passing through the membrane was continuously collected in a clean beaker positioned at the permeate outlet, while the concentrate stream was recirculated within the system. The laboratory-scale cross-flow filtration apparatus employed in this study is illustrated in Fig. 1.

At the end of the filtration period, the total permeate collected was weighed using an analytical balance. The measured permeate mass was subsequently used to calculate membrane permeate flux according to eqn (1), as previously described by Jun *et al.*,^42^1
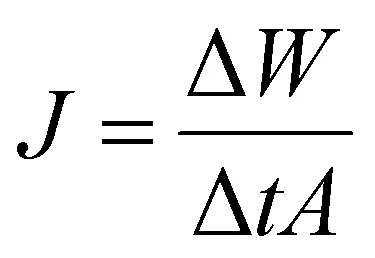
where *J* is the permeate flux (g m^−2^ h^−1^), representing the mass of water permeating through a unit membrane area per unit filtration time; Δ*W* is the mass of permeate collected during the filtration experiment (g); *A* is the effective membrane filtration area (m^2^); and Δ*t* is the filtration time (h). The calculated permeate flux provides a quantitative measure of membrane hydraulic performance after oxidative exposure. An increase in permeate flux relative to the untreated membrane generally indicates deterioration of the polyamide selective layer, resulting from structural damage such as oxidation, cracking, or loss of membrane selectivity. Consequently, permeate flux analysis was used in conjunction with membrane surface characterization to establish the relationship between ClO_2_ exposure conditions, membrane degradation behavior, and changes in membrane integrity.

To ensure consistency among experimental conditions, identical operating parameters, including transmembrane pressure, filtration duration, feed volume, and flow rate, were maintained throughout all experiments. Therefore, differences in permeate flux were attributed primarily to changes in membrane properties induced by ClO_2_ exposure rather than variations in filtration operating conditions. The permeate flux data obtained from this analysis were subsequently correlated with SEM-EDX observations and contact angle measurements to provide a comprehensive evaluation of membrane degradation behavior and surface property changes under the investigated ClO_2_ exposure conditions.

### 2.5. Membrane surface characterization

Following membrane performance evaluation, the effects of ClO_2_ exposure on membrane integrity were further investigated through surface characterization analyses. Morphological changes, elemental composition, and surface wettability were examined to provide complementary evidence of oxidative degradation induced under different ClO_2_ concentrations and solution pH conditions. The characterization techniques employed in this study comprised SEM-EDX and static water contact angle measurements. Together, these analyses enabled qualitative assessment of membrane degradation behavior and changes in surface properties associated with oxidative exposure.

#### 2.5.1. SEM-EDX analysis

SEM-EDX was employed to examine changes in membrane surface morphology and elemental composition following ClO_2_ exposure. SEM analysis is widely used for investigating structural alterations in polymeric membranes because it provides high-resolution visualization of surface features,^43^ while EDX complements morphological observations by identifying variations in elemental composition that may accompany oxidative degradation.^44^ Prior to SEM observation, membrane specimens were cut from the central region of each membrane coupon to minimize edge effects and ensure representative characterization of the exposed surface.^45^ Each specimen was visually inspected, and representative areas free from mechanical damage unrelated to the experimental treatment were selected for imaging. To improve electrical conductivity and reduce charging during electron beam irradiation, the membrane surfaces were coated with a thin platinum layer using a high-purity platinum sputter target before analysis.

Surface morphology and elemental composition were analyzed using a JEOL JSM-6510LA SEM-EDX system (JEOL Ltd., Tokyo, Japan). SEM micrographs were acquired at multiple magnifications to enable observation of membrane surface features over different length scales, ranging from overall surface morphology to localized structural deterioration. When available, an accelerating voltage of 15 kV was employed, which is commonly used for polymeric membrane characterization and provides an appropriate balance between image resolution and analytical depth for EDX measurements.

The SEM images obtained in this study were interpreted qualitatively to compare membrane degradation resulting from exposure to different ClO_2_ concentrations and pH conditions. Particular attention was given to the presence of surface cracking, disruption of the polyamide selective layer, pore exposure, surface irregularities, and other morphological features indicative of oxidative deterioration. Likewise, EDX spectra were used to qualitatively evaluate changes in surface elemental composition associated with membrane oxidation. Quantitative image analyses, including surface roughness measurements, pore size distribution, cross-sectional thickness determination, and image-based statistical analysis, were beyond the scope of the present investigation. Accordingly, SEM–EDX was employed as a complementary characterization technique to support interpretation of the observed changes in membrane hydraulic performance.

#### 2.5.2. Contact angle measurement

The wettability of membrane surfaces before and after ClO_2_ exposure was evaluated by static water contact angle measurements using the sessile drop method.^46^ Surface wettability is an important physicochemical property of polymeric membranes because it reflects changes in surface energy that may arise from oxidation-induced chemical modification and structural degradation.^47^ Variations in contact angle therefore provide useful information regarding alterations in membrane surface characteristics following oxidant exposure.

Contact angle measurements were performed using an OCA 25 optical contact angle analyzer (DataPhysics Instruments GmbH, Germany). Prior to measurement, membrane specimens were allowed to equilibrate under laboratory conditions to minimize the influence of residual moisture. A small droplet of deionized water was carefully deposited onto the membrane surface using the instrument's automated dispensing system, forming a sessile droplet. The contact angle formed between the liquid droplet and the membrane surface was automatically recorded by image analysis software immediately after droplet deposition.

To improve measurement consistency, droplets were placed on representative regions located near the central area of each membrane specimen while avoiding visible defects, folds, scratches, or localized damage generated during membrane handling or oxidative exposure. The measured contact angle values were subsequently used as an indirect indicator of changes in membrane wettability associated with ClO_2_-induced oxidative degradation. In general, an increase in contact angle indicates a more hydrophobic membrane surface, whereas a decrease reflects increased hydrophilicity. Because membrane surface wettability influences interactions between the membrane and aqueous constituents, changes in contact angle also provide useful insight into surface characteristics that may affect fouling propensity. However, it should be emphasized that the present study did not directly evaluate biofouling through microbial adhesion or biofilm formation experiments. Consequently, the contact angle results were interpreted solely in relation to oxidative degradation and associated changes in membrane surface properties rather than as direct measurements of antifouling or biofouling performance.

## 3. Results and discussion

The influence of ClO_2_ exposure on RO membrane integrity was evaluated through complementary analyses of membrane hydraulic performance, surface morphology, and surface wettability. These characterization techniques collectively provide insight into the degradation behavior of TFC polyamide membranes under different oxidant concentrations and pH conditions that are representative of practical RO pretreatment applications. Permeate flux measurements were employed to quantify changes in membrane hydraulic performance following oxidative exposure, while SEM–EDX and contact angle analyses were subsequently used to investigate corresponding alterations in membrane morphology, elemental composition, and surface wettability. Through integrating these analytical approaches, the present study establishes mechanistic relationships between oxidative exposure conditions, membrane degradation behavior, and surface property changes that are relevant to the long-term operation of RO systems. Although direct microbial adhesion or biofilm formation experiments were beyond the scope of this work, the observed changes in membrane integrity provide important implications for biofouling control because membrane surface characteristics strongly influence the initial stages of microbial attachment and subsequent biofilm development.

### 3.1. Changes in permeate flux of RO membranes under different ClO_2_ concentrations and pH conditions

The hydraulic performance of the RO membranes following chlorine dioxide exposure was evaluated using the laboratory-scale cross-flow filtration system. Permeate production was continuously monitored throughout the filtration experiment to determine the influence of ClO_2_ concentration and solution pH on membrane permeability after oxidative treatment. Because oxidative degradation of the polyamide selective layer can modify membrane pore structure, surface chemistry, and transport resistance, permeate flux provides a practical indicator of changes in membrane integrity.

**Fig. 2:**
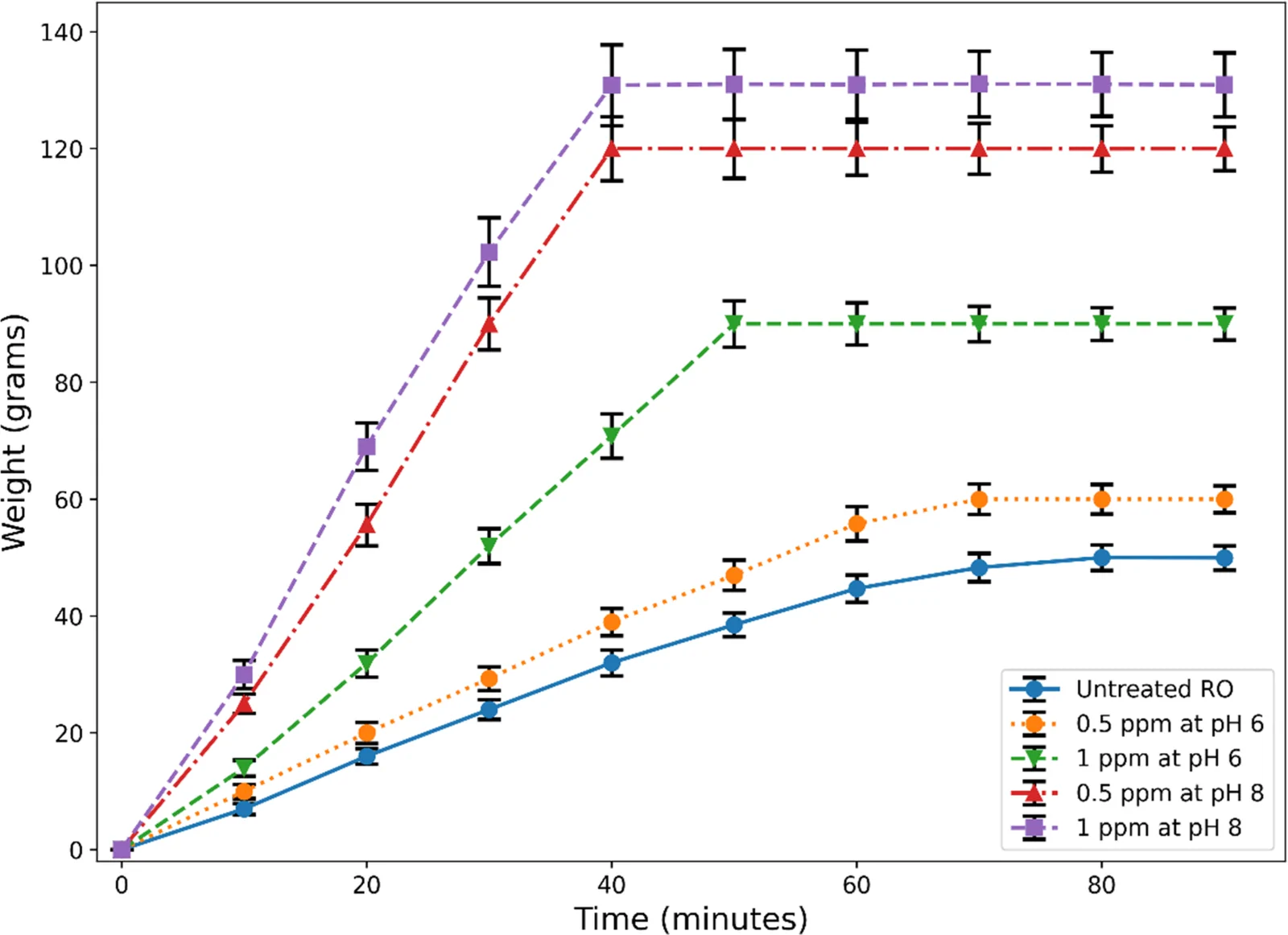
Weight gain of permeate solution collected during cross-flow filtration under different ClO_2_ concentrations and pH conditions.

Fig. 2 illustrates the cumulative permeate weight collected during the filtration experiments for untreated membranes and membranes exposed to different combinations of ClO_2_ concentration and solution pH. For all membrane conditions, permeate weight increased rapidly during the initial stage of filtration before gradually approaching a plateau as the filtration progressed. The untreated RO membrane exhibited the lowest cumulative permeate production throughout the experiment, reflecting the highest hydraulic resistance and the intact structure of the pristine TFC polyamide selective layer. In contrast, membranes exposed to ClO_2_ produced substantially greater permeate volumes, indicating that oxidative exposure increased membrane permeability.

The magnitude of permeate production increased systematically with both increasing ClO_2_ concentration and increasing solution pH. Membranes treated with 0.5 ppm ClO_2_ at pH 6 exhibited only a modest increase in permeate production relative to the untreated membrane, suggesting that oxidative modification of the polyamide layer remained limited under these relatively mild operating conditions. Increasing the oxidant concentration to 1.0 ppm at pH 6 produced a substantially greater increase in cumulative permeate weight, indicating more pronounced degradation of the membrane selective layer. An even larger increase was observed under mildly alkaline conditions (pH 8), where membranes exposed to 0.5 ppm and 1.0 ppm ClO_2_ reached cumulative permeate weights of approximately 120 g and 130 g, respectively, considerably exceeding those obtained under near-neutral conditions.

The earlier stabilization of cumulative permeate weight observed for membranes exposed to higher ClO_2_ concentrations and alkaline conditions should not be interpreted as direct evidence of membrane fouling. Instead, the plateau primarily reflects stabilization of permeate production under the fixed experimental conditions after substantial water transport had occurred. Because the present study was designed to investigate membrane degradation rather than fouling kinetics, Fig. 2 is interpreted principally in terms of changes in membrane permeability resulting from oxidative modification of the polyamide selective layer.

**Fig. 3:**
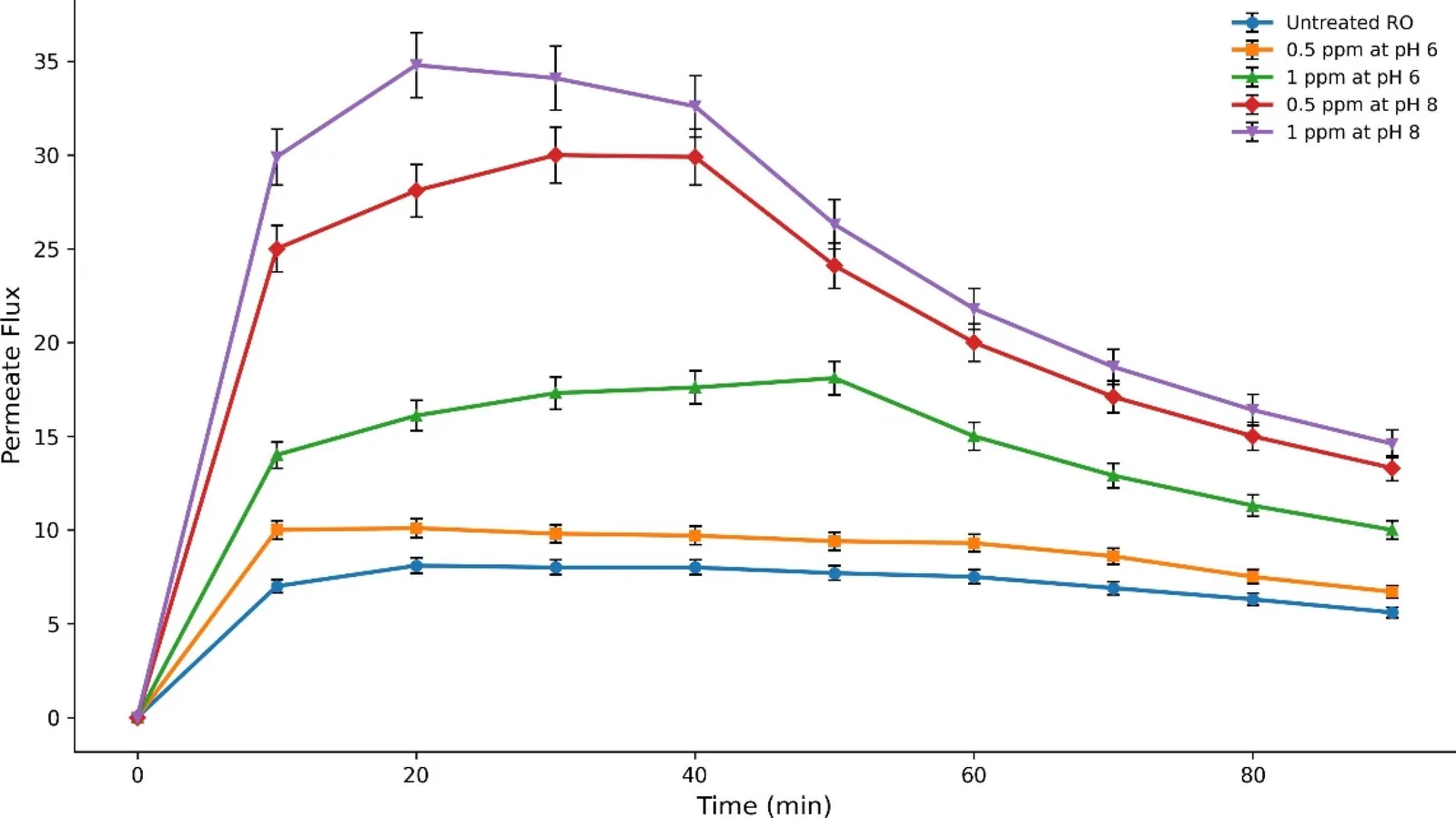
Permeate flux of RO membranes following exposure to different ClO_2_ concentrations and solution pH conditions.

The corresponding permeate flux values calculated using eqn (1) are summarized in Fig. 3. The untreated membrane exhibited the lowest permeate flux of 6.3 g m^−2^ h^−1^, whereas membranes exposed to 0.5 ppm ClO_2_ at pH 6, 1.0 ppm ClO_2_ at pH 6, 0.5 ppm ClO_2_ at pH 8, and 1.0 ppm ClO_2_ at pH 8 exhibited permeate fluxes of approximately 8.6, 18.1, 29.9, and 32.6 g m^−2^ h^−1^, respectively. These results demonstrate a progressive increase in membrane permeability with increasing oxidant concentration and increasing solution pH.

The increase in permeate flux is attributed primarily to oxidative degradation of the TFC polyamide selective layer. Chlorine dioxide is a strong oxidizing agent capable of attacking susceptible functional groups within polyamide membranes, resulting in oxidation of amide linkages, partial chain scission, and disruption of the dense selective layer. As oxidative degradation progresses, defects and enlarged transport pathways may develop within the membrane structure, thereby reducing hydraulic resistance and allowing greater water transport across the membrane. Consequently, the observed increase in permeate flux reflects deterioration of membrane integrity rather than improved membrane performance.

The influence of solution pH was particularly pronounced. Although the standard oxidation potential of ClO_2_ generally decreases with increasing pH according to electrochemical principles, the greater membrane degradation observed under alkaline conditions is consistent with the increased susceptibility of polyamide materials to hydrolytic and oxidative attack. Mildly alkaline conditions can facilitate hydrolysis of amide bonds while simultaneously promoting structural weakening of the selective layer, thereby increasing membrane permeability. Similar observations have been reported by Kim *et al.*,^48^ who demonstrated that membrane permeability increased following oxidative degradation of the polyamide layer. Likewise, Wang *et al.*^49^ reported enlargement of membrane transport pathways following degradation of polyamide structures, leading to increased water permeability and reduced membrane selectivity.

The progressive increase in permeate flux observed in the present study therefore suggests increasing disruption of the selective polyamide layer with increasing ClO_2_ concentration and solution pH. Under the most aggressive exposure condition (1.0 ppm ClO_2_ at pH 8), the membrane exhibited a permeate flux approximately five times greater than that of the untreated membrane. Although higher permeability may initially appear advantageous from an operational perspective, it actually represents deterioration of membrane selectivity and loss of structural integrity. In practical RO applications, excessive increases in permeate flux following oxidant exposure are undesirable because they are frequently accompanied by reduced salt rejection, decreased separation efficiency, and shortened membrane service life.

The present findings are consistent with previous reports describing oxidative degradation of polyamide RO membranes following exposure to chlorine-based oxidants. Kim *et al.*^48^ reported that oxidation of the polyamide selective layer increased membrane permeability through structural damage, while García-Triñanes *et al.*^50^ emphasized that membrane degradation and mineral deposition may occur simultaneously under unfavorable chemical conditions, accelerating long-term membrane deterioration. Similarly, Ahmed and Mohamed^51^ demonstrated that low concentrations of ClO_2_ can effectively support microbial control during RO pretreatment, whereas excessive oxidant exposure may progressively compromise membrane performance through oxidative degradation.

It should be emphasized that the present study did not directly investigate membrane fouling, scaling kinetics, microbial attachment, or biofilm formation. Consequently, increases in permeate flux should not be interpreted as indicators of fouling behavior. Instead, the observed hydraulic changes primarily reflect degradation of membrane structure induced by oxidative exposure. Nevertheless, changes in membrane integrity and surface characteristics have important operational implications because membrane morphology, permeability, and wettability influence the susceptibility of RO membranes to subsequent fouling processes during long-term operation. These relationships are further explored through SEM–EDX and contact angle analyses presented in the following sections.

Among the investigated operating conditions, exposure to 0.5 ppm ClO_2_ at pH 6 produced the smallest increase in permeate flux relative to the untreated membrane, indicating the least severe membrane degradation. This observation supports the premise that conservative ClO_2_ dosing under near-neutral conditions provides a more appropriate balance between effective oxidative disinfection and preservation of membrane integrity. Conversely, higher ClO_2_ concentration combined with mildly alkaline conditions accelerated degradation of the polyamide selective layer, highlighting the importance of optimizing oxidant dosage and operating pH to minimize long-term deterioration of RO membranes.

### 3.2. Surface morphology and elemental characteristics of RO membranes following ClO_2_ exposure

**Fig. 4:**
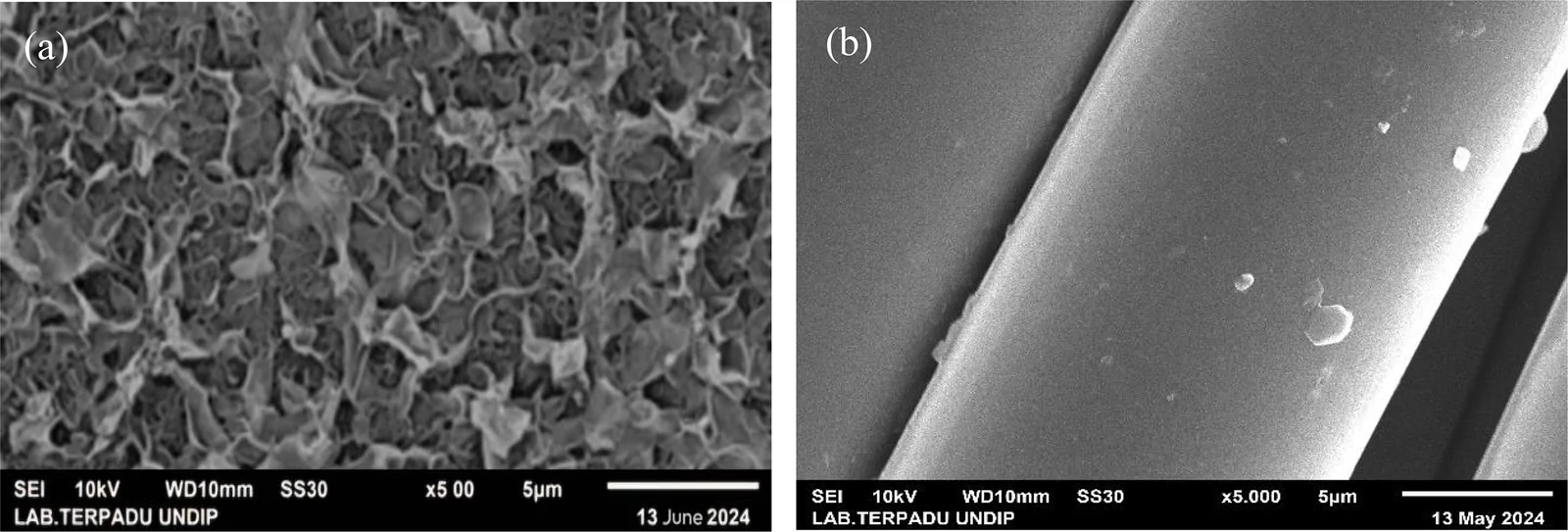
Top layer surfaces: (a) 500× and (b) 5000×.

Fig. 4a and b depict the surface structure of the top layer of the RO membrane before being treated with ClO_2_ using SEM-EDX with (a) 500× magnification, and 5000× magnification. According to Alkhouzaam *et al.*,^52^ figure of untreated RO membrane with 500× magnification, reveals that it is the TFC RO membrane's thin selective layer. It had a solid, smooth surface with few apparent pores. In 5000× magnification, there is only minor stretched and wrinkle. A little amount of foulant is also visible. Polyamide was utilized to construct a thin selective layer, which was principally constituted of a polyamide film formed by interfacial polymerization. This layer had a thick, cross-linked polymer matrix that provides great selectivity by allowing water molecules to pass while inhibiting dissolved salts and impurities. The polyamide layer was extremely thin and has a rough surface, which increases the surface area accessible for water permeability.^51^

In terms of exposure tests to different pH values and ClO_2_ concentrations, the color changes of RO membrane before and after exposure to different ClO_2_ (0.5 ppm and 1 ppm) concentrations and pH values (pH 6 and 8). After exposure, all membranes change color on their respective surfaces. The surface of the RO membrane turned entirely white as the pH valued climbed. Precipitate could cause a whitish appearance in the membrane. It is generated by the interaction of chlorine dioxide with the polyamide membrane, which could yield insoluble byproducts. These byproducts may form a precipitate on the membrane surface.^51^

In contrast, when the pH value of the RO membrane decreased, it turned yellow. The color shift of the RO membrane to yellow can be caused by changes and degradation of the membrane's polyamide structure, resulting in discoloration. Chemical deterioration not only alters the color of the membrane, but it also had a detrimental influence on its performance. The membrane's integrity and selectivity were weakened, resulting in lowered efficiency in rejecting pollutants. Yellowing on RO membranes is mainly caused by fouling or pollution.^53^ The observed membrane degradation can be attributed to oxidative reactions induced by ClO_2_, which are known to affect the polyamide active layer. Oxidative attack may lead to amide bond cleavage, resulting in chain scission and disruption of the membrane structure. This process is consistent with the SEM observations showing surface damage and cracking, which in turn contribute to increased permeate flux due to reduced structural integrity. In addition, the decrease in contact angle suggests an increase in surface hydrophilicity, which may be associated with the introduction of oxygen-containing functional groups, for example carboxyl or hydroxyl groups as a result of oxidation. Previous studies using FTIR and XPS have reported that exposure to chlorine-based oxidants can lead to amide bond cleavage, formation of carboxylic groups, and aromatic ring modification in polyamide membranes. These findings support the proposed mechanism in the present study, although direct spectroscopic evidence was not obtained. While oxidative degradation appears to be the dominant mechanism, it is also possible that under certain conditions, limited cross-linking or structural rearrangement may occur, potentially stabilizing specific regions of the membrane. However, such effects were not dominant in the present study, as indicated by the overall increase in permeability and structural damage.

**Fig. 5:**
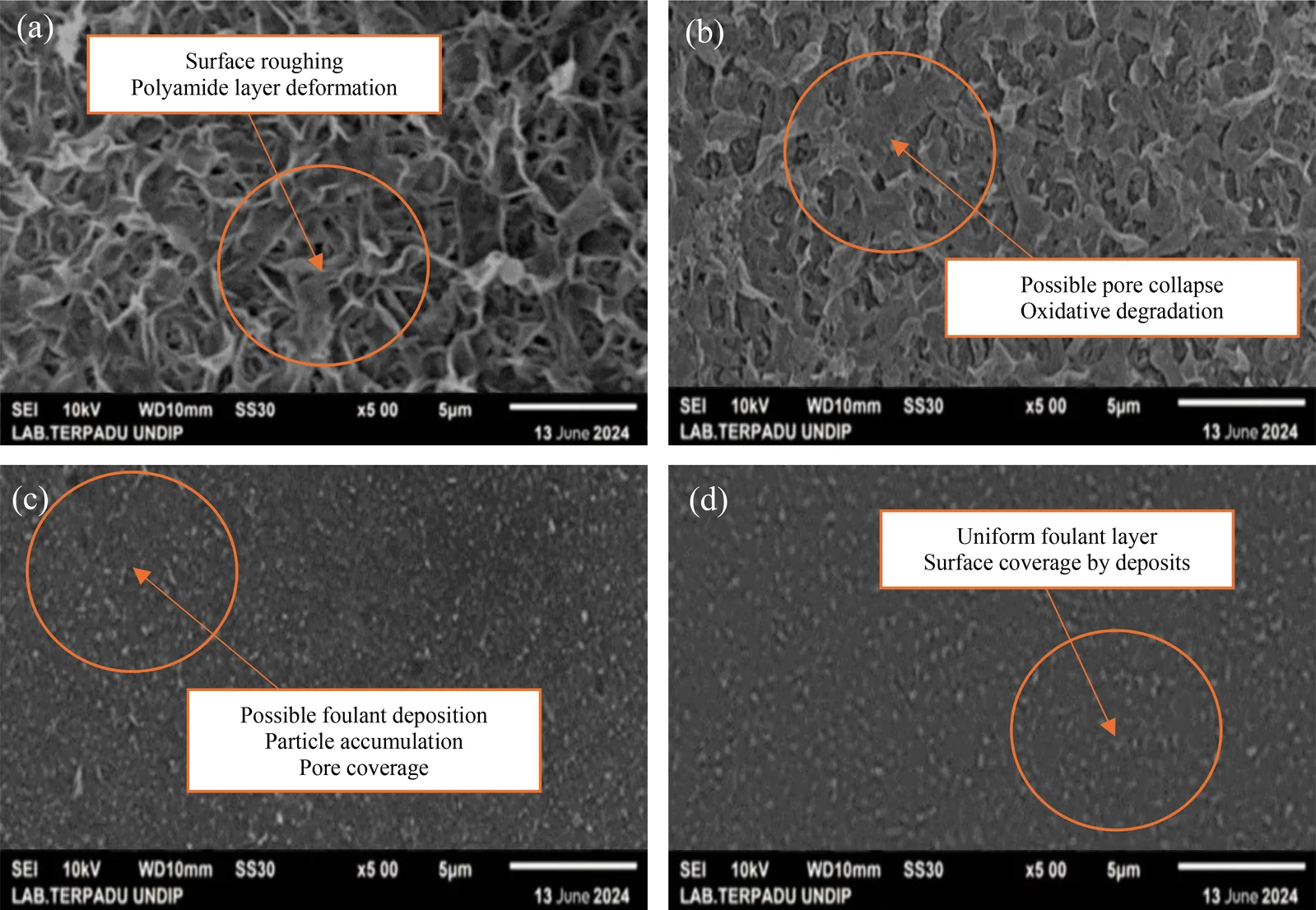
Top layer surfaces: (a) 0.5 ppm ClO_2_ at pH 6, (b) 1 ppm ClO_2_ at pH 6, (c) 0.5 ppm ClO_2_ at pH 8, and (d) 1 ppm ClO_2_ at pH 8 with 500× magnification (arrows and circles indicate regions of potential foulant deposition, pore blockage, and surface structural changes).

The surface structure of the top layer of the RO membrane after treated with ClO_2_ using SEM-EDX with 500× magnification (a) 0.5 ppm ClO_2_ pH 6, (b) 1 ppm ClO_2_ pH 6, (c) 0.5 ppm ClO_2_ pH 8, and (d) 1 ppm ClO_2_ pH 8 can be seen in Fig. 5a–d.

The identification of foulants is based on morphological observation and should be interpreted qualitatively. Fig. 5 shows the surface structure of RO membranes after exposure to ClO_2_ 0.5 ppm and 1 ppm solutions at various pH levels with 500× magnification. SEM observation of untreated RO membrane reveals a visible thin selective layer with there was a slight stretch and wrinkle. A little amount of foulant is also visible. The RO membrane exposed to 0.5 ppm ClO_2_ concentration at pH 6 showed a high concentration of foulants adhering to the thin selective layer. Both stretch and deformation were occurred in the layer. This membrane was still cohesive and was not severely damaged.

The SEM observations reveal a progressive increase in surface damage with increasing ClO_2_ concentration and pH. At 0.5 ppm and pH 6, only minor surface wrinkling and deformation are observed, whereas at higher pH and concentration (1 ppm, pH 8), severe structural disruption, including cracking and exposure of the underlying support layer, becomes evident. This membrane variation depicts the construction of a reinforcing nonwoven backing layer that serves as extra structural support. This indicates that the thin selective layer and porous polymer substrate layer have deteriorated. According to Gao *et al.*,^54^ a nonwoven backing layer with high porosity promotes water flow while minimizing resistance and resisting degradation from chemicals often encountered in water treatment operations. ClO_2_ is an extremely powerful oxidizing agent, while polyamide membrane known to be easily damaged by oxidants. In aqueous systems, chlorine dioxide can undergo partial decomposition depending on pH, temperature, and exposure time. The primary decomposition products include ClO_2_^−^ and ClO_3_^−^, with minor formation of chloride (Cl^−^). The distribution of these species is influenced by pH, and they may also contribute to oxidative interactions with the polyamide layer. Therefore, membrane degradation observed in this study may result from combined effects of ClO_2_ and its secondary chlorine-containing species. Exposing polyamides to ClO_2_ at high pH conditions can significantly degrade their structure. As the amide bonds were oxidized or hydrolyzed, the hydrogen bonds that keep the polymer chains together were broken. The deterioration of the polymer matrix would result in a loss of tensile strength, stiffness, and other mechanical qualities. Surface deterioration or fragmentation of the polyamide layer may occur when the chemical structure degrades.^55^

The degradation in this membrane was severe, until the reinforcing nonwoven backing layer structure is clearly seen. Foulants were also present in this membrane and both stretching, and deformation is also more visible in this membrane. While the RO membrane that exposed to 0.5 ppm ClO_2_ concentration at pH 6 is still cohesive and is not severely damaged even though it shows high concentration of foulants in the membrane.

In the RO membrane exposed to 1 ppm ClO_2_ concentration at pH 6 stretching and deformation are more visible in this membrane. Along with the increase in magnification used to observe the membrane, the degradation had grown increasingly noticeable. This membrane variation removed the polyamide structure's harsh ridges and valleys, resulting in a porous surface. According to Alkhouzaam *et al.*,^52^ this was most likely the porous polymer substrate layer (polysulfone layer) that serves as a support layer beneath the polyamide structure. The visibility of the porous polymer substrate layer reveals that the thin selective layer TFC membrane has disintegrated as a result of exposure to 1 ppm of ClO_2_ at pH 6.

The thin selective and supporting layers entirely disintegrate, revealing the backing layer. In the RO membrane exposed to 0.5 ppm ClO_2_ concentration at pH 8, major creases, cracks, and deformations of this RO membrane were obvious. The stretching and degradation of the layer might cause an increase in permeate flux. The degradation in this membrane was severe, until the reinforcing nonwoven backing layer structure was clearly seen. Foulants were also present in this membrane.^56^ Although the analysis is qualitative, the consistent progression of these features suggests increasing degradation severity, likely driven by oxidation and hydrolysis of the polyamide layer.

According to previous research, exposure to ClO_2_ could cause deformation, stretch and wrinkle in RO membranes due to a variety of chemical and physical reactions. These include:

1. Chemical degradation

ClO_2_ had the potential to significantly oxidize the polymer chains in the RO membrane. This oxidation could cause the polymeric structure to break down, resulting in changes in mechanical characteristics. ClO_2_, when paired with high pH, may increase the hydrolysis of the polyamide layer in the RO membrane. This could further deteriorate the material by disrupting amide bonds in the polymer chain.^57^

2. Structural alteration

ClO_2_'s oxidative activity could break down polymer chains within the membrane. This breakdown of the polymer network could result in a loss of structural integrity, making the material less stable and prone to deformation.^56^

3. Physical changes

ClO_2_'s chemical attack might cause membrane swelling owing to water and solvent absorption. This swelling could cause internal tension inside the membrane, resulting in physical deformities such as stretching and wrinkling. As some sections of the membrane deteriorate faster than others, tension and compression can form in specific places. Force imbalance across the membrane can cause stretching in particular areas and wrinkling in others.^52^

## 4. Surface layer effects

Oxidation and hydrolysis had the potential to enhance membrane surface roughness. This variation in surface characteristics could contribute to an unequal distribution of stresses, resulting in noticeable wrinkles and strains. The membrane's elastic characteristics may also be lost as a result of chemical structural degradation. This decrease of elasticity renders the membrane more susceptible to persistent deformation when subjected to mechanical stress.^56^ In previous research, RO membranes are similar in terms of fully aromatic polyamide based on interfacial polymerization of 1,3-diaminobenzene(*m*-phenylenediamine) (MPD) and 1,3,5-benzenetricarbonyl trichloride (trimesoyl chloride) (TMC). The resulting polyamide had a highly cross-linked, aromatic structure providing high salt rejection and water permeability.^51^

**Fig. 6:**
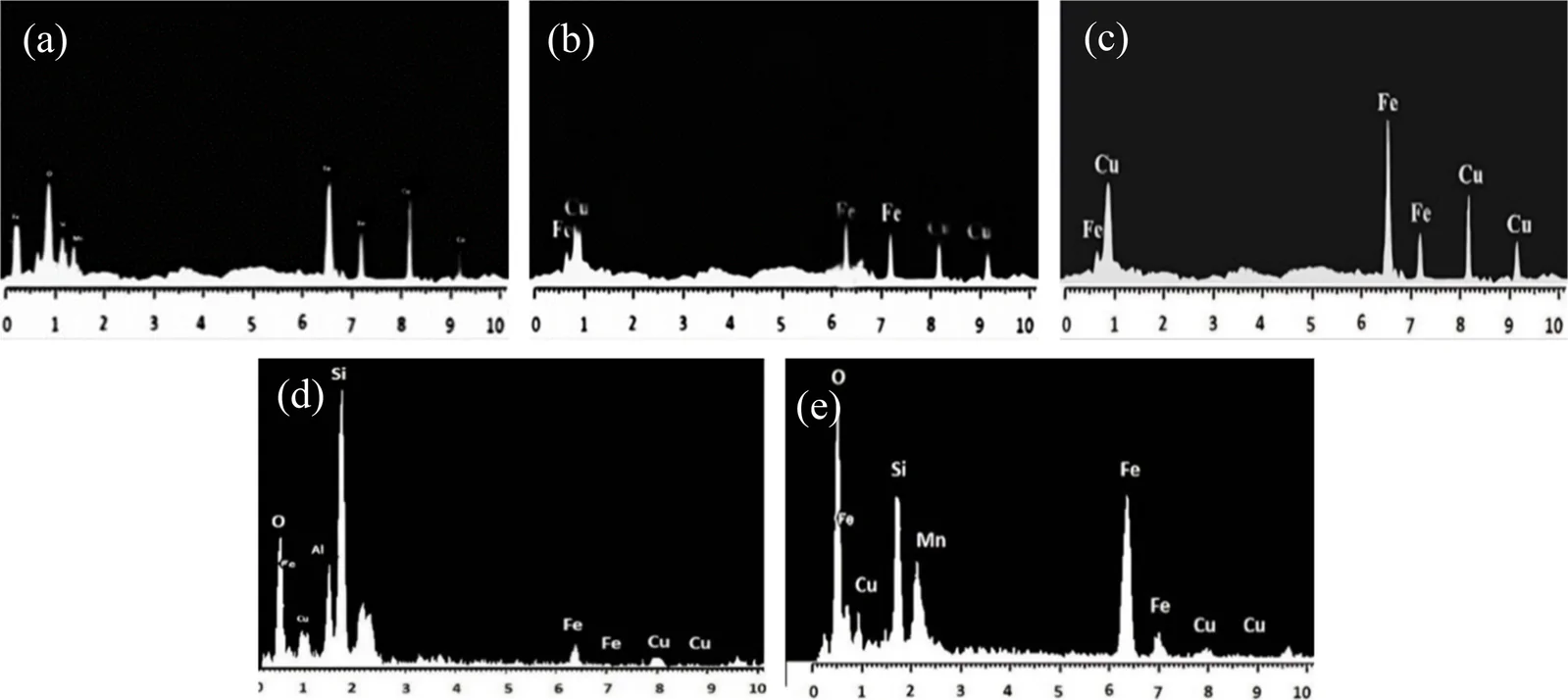
EDX: (a) untreated RO, (b) 0.5 ppm at pH 6, (c) 1 ppm at pH 6, (d) 0.5 ppm at pH 8, and (e) 1 ppm at pH 8.

In addition to SEM, Energy Dispersive X-ray (EDX) was also conducted to identify the elemental composition of the membrane surface before treated with ClO_2_, as shown in Fig. 6. The EDX analysis further supports this interpretation, as changes in elemental composition, such as an increase in oxygen content relative to carbon may indicate oxidative modification of the membrane surface. Such compositional shifts are commonly associated with polymer oxidation and degradation processes. While to identify changes in the elemental composition of the membrane surface caused by ClO_2_ exposure providing complementary chemical information to support the morphological analysis can be seen in Fig. 6.

The presence of Fe compounds on the membrane may be related to the quality of tap water utilized in this research, which still contains Fe. These Fe and Cu compounds tended to cause fouling of the RO membranes. According to Melliti *et al.*,^58^ iron in water can oxidize and precipitate as ferric hydroxide due to the presence of oxygen, which remains to the membrane surface and causes fouling. Oxygen can cause Fe^2+^ to convert to Fe^3+^ (which is less soluble). This can cause the production of iron hydroxides (Fe(OH)_3_), which could result in deposits on the membrane surface (eqn (2)):

2 4Fe^2+^ + O_2_ + 6H_2_O → 4(Fe(OH)_3_)

These deposits reduce water flow, decreasing the membrane's efficiency and longevity. Copper develops when copper ions precipitate as copper hydroxide (Cu(OH)_2_) from the feed water and create deposits on the membrane surface (eqn (3)).

3 Cu^2+^ + 2OH^−^ → Cu(OH)_2_

Cu could interact with other contaminant like organic matter and later on will form complex deposits, that further exacerbate fouling.^50^ The EDX graph of the RO membrane subjected to 0.5 ppm ClO_2_ at pH 8 showed amount of Si, Al, and O compound. Si, Al, and O compounds can foul RO membranes in the same way as Fe and Cu compounds could. In water, silica would react to form mono-silica acid (eqn (4)).

4 SiO_2_ + 2H_2_O → Si(OH)_4_

Si(OH)_4_ or silicic acid can precipitate and create a scale on membrane surfaces. This sort of fouling was very difficult to control and remove because of its high adhesion and low solubility.^59^ O compounds were found in this RO membrane variation. Oxygen compounds could have been generated from membrane components such as polyamide or from the membrane that was broken down or oxidized by water containing a strong oxidizer (ClO_2_). This process can generate oxygen compounds, which can subsequently be identified by EDX. O compounds can produce fouling in an indirect manner. For example, oxygen may react with some dissolved chemicals in water, resulting in the development of oxides or biofilms that are important pollutants. According to Maeda,^60^ oxygen could increase the growth of aerobic microorganisms on RO membranes, resulting in biofouling. Additionally, if dissolved metals in the feed water, such as Fe and Mn are in contact with oxygen compound, it will forming insoluble oxides that precipitate and buildup on the membrane surface, resulting in inorganic fouling.

The EDX graph of the RO membrane subjected to 1 ppm ClO_2_ at pH 8 showed O, Si, Mn, and Fe compound in the RO membrane. The preceding paragraph discussed how O, Si, and Fe compounds might cause fouling on the RO membrane. Mn compounds were still often found in tap water. Mn is soluble (Mn^2+^) in low pH; therefore, precipitation is unlikely. However, in high pH, Mn is more likely to precipitate as manganese hydroxides (Mn(OH)_2_). This chemical could enhance fouling potential by reacting with organic molecules, resulting in complexes that deposit on the membrane surface and dramatically decrease water flow. When Mn interacts with organic contaminants including humic acid (HA), sodium alginate (SA), and combinations of different organic compounds, it could aggravate membrane fouling.^54^ Mn^2+^ could oxidize to Mn^4+^ in water when exposed to air or an oxidizing agent (ClO_2_). This oxidation process produces insoluble manganese oxides (MnO_2_), which could precipitate. Interestingly, no significant chlorine signal was detected in the EDX spectra after ClO_2_ exposure. Although previous studies have reported possible chlorine incorporation into polyamide membranes under oxidative conditions, chlorine dioxide primarily acts as a selective oxidant rather than a direct chlorinating agent. Moreover, EDX analysis has limited sensitivity for detecting low concentrations of surface-bound chlorine, especially when chlorination occurs only within a very thin surface layer. In addition, the rinsing step after soaking may have removed loosely associated chlorine-containing species, resulting in undetectable chlorine levels in the analysis. The SEM analysis presented here provides qualitative evidence of membrane degradation, including surface deformation, cracking, and exposure of the underlying support layer. While these observations indicate progressive structural damage with increasing ClO_2_ concentration and pH, no quantitative metrics (such as roughness parameters or pore size distribution) were extracted from the images. Nevertheless, the consistent appearance of morphological features across different treatment conditions supports the proposed degradation mechanisms, particularly oxidation and hydrolysis of the polyamide layer.

### 4.1. Hydrophilicity changes of the RO membrane treated with ClO_2_ (contact angle)

**Fig. 7:**
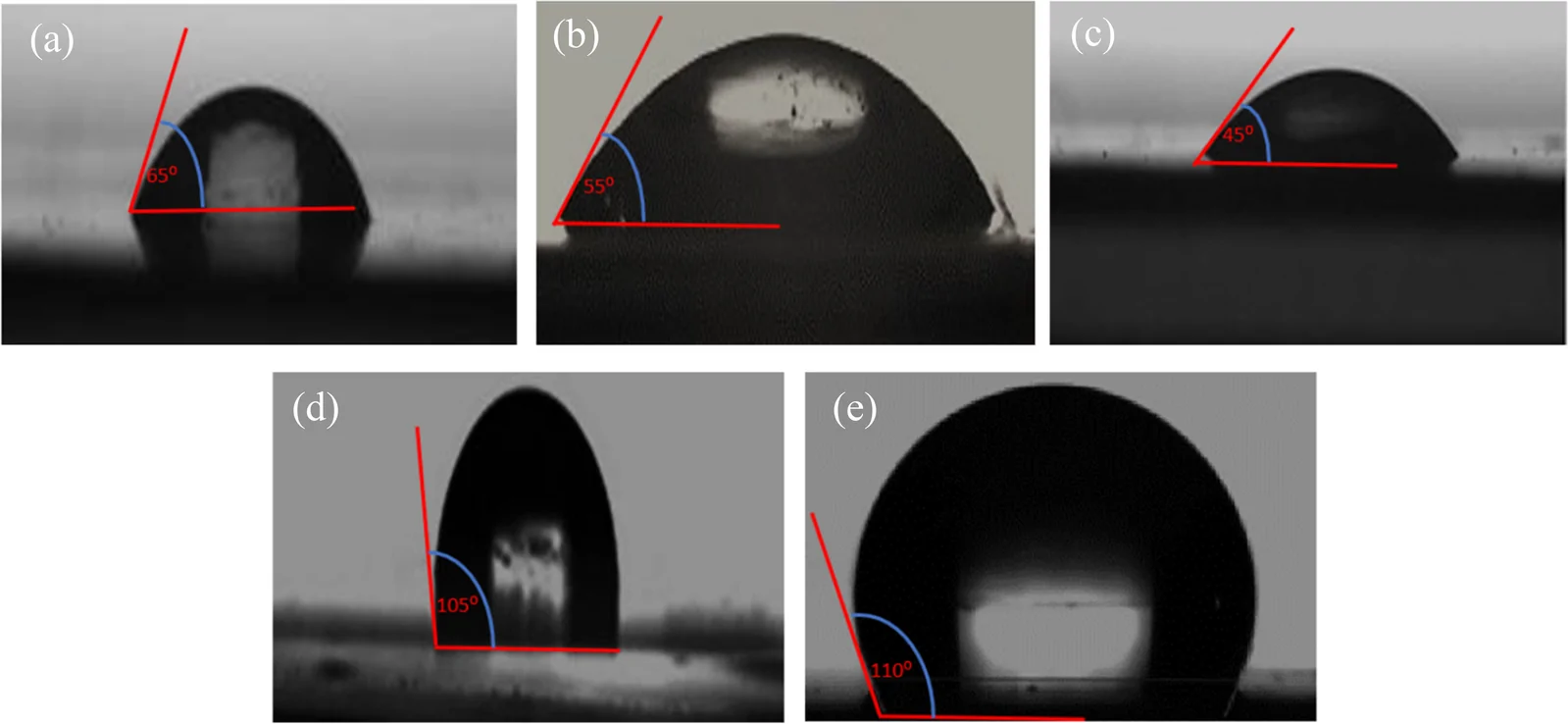
Contact angle: (a) untreated RO, (b) 0.5 ppm ClO_2_ at pH 6, (c) 1 ppm ClO_2_ at pH 6, (d) 0.5 ppm ClO_2_ at pH 8, and (e) 1 ppm ClO_2_ at pH 8.

The observed decrease in contact angle suggests an increase in membrane hydrophilicity following ClO_2_ exposure. However, due to the limited number of measurements, these differences should be interpreted cautiously and may indicate a trend rather than a statistically significant change. Fig. 7 depicts how the contact angles data and figure of contact angles collected used the watered sessile dropped method was before and after the RO membranes was exposed to ClO_2_. The contact angle indicates the hydrophilicity and hydrophobicity of the studied membrane. Overall, hydrophilicity rose as the contact angle lowers. Low contact angle implies high watered affinity, and any changed in the contact angle might suggested increased fouling development. A rougher membrane, with a bigger effective surface area and/or a more hydrophilic membrane, had a higher permeate flowed than an otherwise equal surface property. Basically, the polyamide thin selective layer in TFC RO membranes was known have been hydrophilic. This means that this layer had a high affinity for watered, which helps in the process of separating watered from contaminants. The hydrophilic nature helps watered to penetrate more easily through the membrane, while contaminants such as salts, ions, and large organic molecules were rejected and discharged into the permeate stream. Based on Fig. 7 the contact angle of untreated RO membrane was 650.

The contact angle of RO membrane after exposure of 0.5 ppm ClO_2_ at pH 6 decreases from 650 to 550. This indicates that 0.5 ppm of ClO_2_ at pH 6 makes the RO membrane surface more hydrophilic. Contact angle of RO membrane after exposure of 1 ppm ClO_2_ at pH 6 decrease from 650 to 450. This means that chlorine dioxide remains efficient at concentrations of 1.0 ppm or lower at lower pH, and membranes were shown have been highly permeable to ClO_2_.^49^ The contact angle of RO membrane after exposure of 0.5 ppm ClO_2_ at pH 8 decreases from 650 to 1050 and the contact angle of RO membrane after exposure of 0.5 ppm ClO_2_ at pH 8 decreases from 1050 to 1100. This signifies that the RO membrane is hydrophobic since its contact angle was larger than 90°. Although prior studies revealed that smaller concentrations of ClO_2_ were extremely permeable, as the pH was increased, the RO membrane was shown to be intolerant to ClO_2_ at high pH values.

Previous studies have shown that at low pH (acid), amide groups on polyamides may go through protonation (the addition of H^+^ ions), increasing hydrophilic characteristics due to enhanced contact with water *via* hydrogen bonds. At high pH (base), the amide group on polyamide can be hydrolyzed, resulting in carboxylic acids and amines. This hydrolysis could attack the membrane structure and decrease its hydrophilicity.^56^ Membrane treated with 0.5 and 1 ppm of ClO_2_ at pH 8. According to prior studied, membrane treated with 0.5 and 1 ppm of ClO_2_ at pH 8 has a contact angle value identical to the polyester layer: more than 900 61. Therefore, these considerations prove that ClO_2_ at high pH may degrade the membrane and removed the polyamide and polysulfone layer of the membrane.

### 4.2. Analysis of optimal ClO_2_ concentration and pH value for RO membrane

Based on the RO membrane performance analysis yields same results as the RO membrane surface characteristics analysis. The RO membrane sample with a ClO_2_ concentration of 0.5 ppm at pH 8 had the highest permeate flux and hence the greatest fouling potential. The observed increase in permeate flux should be interpreted with caution. Instead of directly indicates fouling, the elevated flux may be attributed to membrane degradation, such as enlargement of pore structure or partial loss of the selective polyamide layer. This interpretation is supported by SEM observations showing surface deformation and damage, as well as contact angle results indicates changes in surface hydrophilicity. pH levels in RO systems had a substantial impact on permeate flux. Polyamide could undergo hydrolysis at low pH (acidic), causing membrane degradation and a reduction in permeate flow. At high pH (alkaline), polyamide could hydrolyzed or degraded, resulting in reduced permeate flux and increased ion permeability. Organic fouling or inorganic fouling can both occurred, which could lead to fouling.^54^

According to previous research from Kim *et al.*^48^ stated that the permeate flux of the membrane upon exposure rose as the pH value increased. This happens when the polysulfone layer behind the polyamide was exposed, causing the pore size to increase. When polyamide was exposed, the pore size increases. As a result, water may readily flow through the polysulfone layer, resulting in a permeate flux approximately 24 times greater than the unexposed one. Although the intrinsic redox potential of chlorine dioxide does not increase with pH, alkaline conditions may enhance membrane susceptibility to oxidative and hydrolytic attack due to increased hydroxide ion activity and structural weakening of the polyamide layer. As the pH value increased, the pore size of the RO membrane increases owing to polyamide membrane breakdown. The larger pore size of the RO membrane causes the permeate flux to decrease faster. The formation of an irreversible fouling layer may began with pore blockage of microscopic particles, followed by intense contact of the fouling layer with dissolved materials, and eventually by fouling layer compaction due to the drag force of permeation flow.^49^ Some ions in water are more prone to produce precipitates that could adhere to the membrane surface when the pH is elevated. Precipitate on the membrane surface could significantly lowered permeate flux because it clogged the membrane pores, reducing water flow. Scaling was a type of fouling in which precipitates develop on the membrane surface. This indicates that the membrane was fouling. Fouling was also commonly indicated by a large dropped in permeate flow.

Analysis of RO membrane surface characteristics using SEM with various magnifications in this study, the most optimal ClO_2_ concentration and pH value for RO membranes was 0.5 ppm of ClO_2_ at pH 6 since the membrane was least affected by ClO_2_. It was not suggested to treat the RO membrane to 0.5 ppm ClO_2_ concentration at pH 8 and 1 ppm ClO_2_ concentration at pH 8 because the ClO_2_ had a significant effect on the RO membrane. At 500× magnification, RO membranes exposed to 0.5 ppm ClO_2_ at pH 6 were identical to untreated membranes. The membrane's rough ridges and valley structure depict the polyamide membrane surface. This indicates that ClO_2_ does not destroy the RO membrane in that particular version. For RO membranes exposed to ClO_2_ 0.5 ppm at pH 8 and ClO_2_ 1 ppm at pH 8, the harsh ridges and valley's structure indicates the thin selective layer dissolved, revealing a new surface with many holes. This was the polyester layer, which serves for providing greater structural support. According to prior studied, exposing polyamides to ClO_2_ at high pH levels could dramatically broke down their structure. As the amide bonds oxidize or hydrolyze, the hydrogen bonds that hold the polymer chains together break. The broke down of the polymer matrix causes a decrease of tensile strength, stiffness, and other mechanical properties. When the chemical structure weakens, the polyamide layer's surface could deteriorate or fragment.^62^

SEM at 500× magnification of a RO membrane exposed to 0.5 ppm ClO_2_ at pH 6 reveals a significant concentration of foulants clinging to the thin selective layer. In the layer, stretch and deformation occur simultaneously. This membrane remains cohesive and was not badly damaged. The RO membrane subjected to 0.5 ppm ClO_2_ concentration at pH 8 showed significant creases, fractures, and deformations. The stretching and degradation of the layer may produce an increase in permeate flux. This membrane's weakening was extreme, revealing the reinforcing nonwoven backing layer structure. Foulants were also found in this membrane. Stretching and wrinkling in RO membranes could be both a cause and a result of fouling.

These deformations enhance the membrane's susceptibility to fouling by providing more surface area and microenvironments for foulants to cling to, whereas fouling itself could cause mechanical stress, resulting in stretching and wrinkles. Particulates could become lodged increases and strained regions, resulting in greater fouling rates. Wrinkled surfaces created opportunities for biofilm formation, exacerbating fouling problems.^63^ Certain biofouling byproducts, such as organic acids, may also damage the membrane material, causing it to weaken and then stretch or wrinkle. The accumulation of foulants on the membrane surface can cause physical stress, which contributes to mechanical deformation.^64^ Oxidation and hydrolysis have the potential to enhance membrane surface roughness. This variation in surface characteristics can contribute to an unequal distribution of stresses, resulting in noticeable wrinkles and strains. The membrane's elastic characteristics may also be lost as a result of chemical structural degradation. This decrease of elasticity renders the membrane more susceptible to persistent deformation when subjected to mechanical stress.^56^

Analysis of hydrophilicity changes of the RO membrane with contact angle also got the same result with the analysis of RO membrane surface characteristics using SEM, that 0.5 ppm of ClO_2_ at pH 6 was the optimal variation to be use in RO membrane. Based on the Fig. 7, RO membrane after exposure of 0.5 ppm ClO_2_at pH 6 decrease from the untreated RO membrane (650 to 550). This indicates that 0.5 ppm of ClO_2_ at pH 6 makes the RO membrane surface more hydrophilic. The contact angle of RO membrane after exposure of 0.5 ppm ClO_2_ at pH 8 decrease from 650 to 1050 and the contact angle of RO membrane after exposure of 0.5 ppm ClO_2_ at pH 8 decrease from 1050 to 1100. This signifies that the RO membrane is hydrophobic since its contact angle is larger than 90°. According to prior study, membrane treated with 0.5 and 1 ppm of ClO_2_ at pH 8 has a contact angle value identical to the polyester layer: more than 900.^61^ Therefore, these considerations prove that ClO_2_ at high pH may degrade the membrane and remove the polyamide and polysulfone layer of the membrane. This means that chlorine dioxide remains efficient at concentrations of 1 ppm or lower at lower pH, and membranes were shown to be highly permeable to ClO_2_.^49^ Previous study has shown that at low pH (acid), amide groups on polyamides may go through protonation (the addition of H^+^ ions), increasing hydrophilic characteristics due to enhanced contact with water *via* hydrogen bonds. At high pH (base), the amide group on polyamide can be hydrolyzed, resulting in carboxylic acids and amines. This hydrolysis could attack the membrane structure and decrease its hydrophilicity.^56^

To facilitate an integrated interpretation of the experimental results, Table 3 summarizes the combined observations obtained from permeate flux measurements, SEM–EDX characterization, and contact angle analysis under the investigated ClO_2_ concentrations and pH conditions. The table highlights the progressive changes in membrane morphology, elemental composition, wettability, and transport performance, thereby providing a comprehensive overview of membrane degradation behavior and its implications for membrane integrity and potential biofouling control.

**Table 3:** Integrated analysis of membrane properties under different conditions

Membrane condition	Permeate flux	SEM observation	SEM-EDX observation	Contact angle behavior	Overall interpretation
Untreated	Lowest	Intact ridge and valley morphology	Native elemental composition	Reference wettability	Intact TFC membrane
0.5 ppm ClO_2_, pH 6	Slight increase	Minor surface alteration	Limited elemental deposits	Increased hydrophilicity	Lowest degradation
1.0 ppm ClO_2_, pH 6	Moderate increase	Initial structural disruption	Greater oxidation-related changes	Moderate wettability change	Moderate degradation
0.5 ppm ClO_2_, pH 8	High	Cracks and deformation	Mineral deposition (Fe, Si, O, *etc.*)	Increased hydrophobicity	Severe degradation
1.0 ppm ClO_2_, pH 8	Highest	Extensive collapse of selective layer	Pronounced elemental deposits	Highest hydrophobicity	Most severe degradation

SEM and contact angle analyses provide valuable insights into morphological changes and surface properties, it should be acknowledged that this study does not include molecular-level characterization of the membrane. As such, direct evidence of chemical bond alterations within the polyamide layer is not available. The interpretation of oxidative degradation is therefore based on indirect indicators, including surface damage, structural deformation, and changes in hydrophilicity. These observations are consistent with previously reported mechanisms of polyamide oxidation by chlorine-based oxidants. Nevertheless, further investigation using advanced spectroscopic techniques such as Fourier-transform infrared spectroscopy (FTIR)^65^ or X-ray photoelectron spectroscopy (XPS)^66^ is recommended to provide more definitive insights into chemical transformations and bond cleavage mechanisms.

A clear relationship can be observed between membrane structural changes and performance indicators. Under higher ClO_2_ concentrations and alkaline conditions, SEM images reveal significant surface damage, including cracking and deformation, which corresponds to an increase in permeate flux. This suggests that the enhanced permeability is primarily due to structural degradation of the membrane rather than improved filtration performance. Furthermore, the decrease in contact angle indicates increased hydrophilicity, which may be associated with chemical modification of the membrane surface. These combined observations highlight that membrane degradation plays a critical role in altering both transport properties and surface characteristics. However, further studies under continuous operation and realistic feed conditions are required to validate these findings.

Unlike studies focusing on advanced membrane materials such as thin-film nanocomposite membranes designed for improved chlorine resistance, this work emphasizes the behavior of commercially available TFC polyamide membranes under realistic oxidant exposure conditions. This approach provides practical insights into how existing membrane systems respond to ClO_2_ dosing strategies, which is critical for current water treatment operations where membrane replacement is not always immediately feasible.

## 5. Conclusions

The performance and characteristics of RO membranes are influenced by ClO_2_ concentration and pH. Higher pH levels cause degradation of the thin selective and supporting layers, especially at 0.5 and 1 ppm ClO_2_, resulting in increased permeate flux (up to 32.6 g m^−2^ h^−1^) but also irreversible fouling. The optimal condition to reduce fouling occurs at 0.5 ppm ClO_2_ with pH 6. Surface analysis showed structural damage and varying foulant attachment, while contact angle measurements indicate that higher pH increases hydrophobicity, reducing water affinity. From an engineering perspective, the application of ClO_2_ in RO systems should be carefully optimized to balance disinfection performance and membrane durability. While ClO_2_ is effective in controlling microbial activity, excessive exposure may accelerate oxidative degradation of the polyamide layer, as evidenced by increased permeability and structural damage. Based on the findings of this study, ClO_2_ concentrations around 0.5 ppm under near-neutral pH conditions (pH ≈ 6) are suggested as a more stable operating range, as they minimize severe membrane degradation while maintaining acceptable performance. In contrast, higher pH (pH 8) and increased ClO_2_ concentrations (1 ppm) were associated with significant structural damage and should be avoided for long-term operation. These results highlight a critical trade-off: while higher oxidant doses may enhance disinfection, they may also reduce membrane lifespan, potentially leading to increased operational costs due to more frequent membrane replacement or cleaning. Therefore, optimization of dosing strategy is essential to achieve a balance between biofouling control and membrane integrity. From a cost-effectiveness perspective, the use of ClO_2_ should be evaluated not only based on its disinfection efficiency but also on its long-term impact on membrane durability and system maintenance requirements. Alternative strategies, including the use of less aggressive oxidants, hybrid pretreatment systems, or the integration of antiscalants and biofouling control agents, may offer more sustainable solutions depending on the feed water characteristics. In addition, the development of more oxidation-resistant membrane materials or surface modifications may help improve system resilience under oxidant exposure. It should be noted that this study was conducted under controlled laboratory conditions using tap water and batch soaking experiments. As such, the results may not fully represent the complexity of real-world RO systems, where continuous flow, pressure, and diverse foulants are present. Future studies should focus on long-term continuous operation, pilot-scale validation, and testing under realistic feed water conditions to better assess the applicability of these findings in full-scale systems.

## Author contributions

The authors have significantly contributed to the development and the writing of this article. Isni Arliyani: data curation, formal analysis, funding acquisition, investigation, and writing – original draft. Hilmi Iqlima Khoirunnisa: software, visualization, and writing – review & editing. Heri Septya Kusuma: resources, supervision, and writing – review & editing. Muhammad Roil Bilad: conceptualization, methodology, and writing – review & editing. Abdulfatah Abdu Yusuf: project administration, formal analysis, and writing – review & editing. Muhammad Imam Ammarullah: project administration, validation, and writing – review & editing.

## Conflicts of interest

The authors declare that they have no known competing financial interests or personal relationships that could have appeared to influence the work reported in this paper. The authors declare no conflict of interest.

## Nomenclature

### Abbreviations

EDX: Energy dispersive X-ray
FTIR: Fourier-transform infrared spectroscopy
HA: Humic acid
HAA: Haloacetic acid
MDP: *M*-Phenylenediamine
RO: Reverse osmosis
SA: Sodium alginate
SEM: Scanning electron microscopy
SEM-EDX: Scanning electron microscopy coupled with energy-dispersive X-ray spectroscopy
TFC: Thin-film composite
TMC: Trimesoyl chloride
THM: Trihalomethane
XPS: X-ray photoelectron spectroscopy

### Chemical symbols

Cl^−^: Chloride
ClO_2_: Chlorine dioxide
ClO_2_^−^: Chlorite
ClO_3_^−^: Chlorate
Cu(OH)_2_: Copper hydroxide
Fe(OH)_3_: Iron hydroxide
KH_2_PO_4_: Potassium dihydrogen phosphate
K_2_HPO_4_: Dipotassium hydrogen phosphate
MnO_2_: Manganese oxides
Mn(OH)_2_: Manganese hydroxide
NaHSO_3_: Sodium bisulfite
NaOH: Sodium hydroxide pellets
Si(OH)_4_: Silicic acid

### Mathematical variables

A
: Effective membrane filtration area (m^2^)
J
: Permeate flux (g m^−2^ h^−1^)
Δ*t*: Filtration time (h)
Δ*W*: Permeate mass collected (g)

## Data Availability

All data generated or analyzed during this study are included in this published article. No additional datasets were generated or analyzed beyond the contents of the manuscript.
